# Development of Biopolymer and Conducting Polymer-Based Optical Sensors for Heavy Metal Ion Detection

**DOI:** 10.3390/molecules25112548

**Published:** 2020-05-30

**Authors:** Nur Syahira Md Ramdzan, Yap Wing Fen, Nur Ain Asyiqin Anas, Nur Alia Sheh Omar, Silvan Saleviter

**Affiliations:** 1 Department of Physics, Faculty of Science, Universiti Putra Malaysia, Serdang 43400, Malaysia; nursyahira.upm@gmail.com; 2 Functional Devices Laboratory, Institute of Advanced Technology, Universiti Putra Malaysia, Serdang 43400, Malaysia; nurainanas.upm@gmail.com (N.A.A.A.); nuralia.upm@gmail.com (N.A.S.O.); silvansaleviter94@gmail.com (S.S.)

**Keywords:** biopolymer, conducting polymer, heavy metal ions, optical sensors, surface plasmon resonance

## Abstract

Great efforts have been devoted to the invention of environmental sensors as the amount of water pollution has increased in recent decades. Chitosan, cellulose and nanocrystalline cellulose are examples of biopolymers that have been intensively studied due to their potential applications, particularly as sensors. Furthermore, the rapid use of conducting polymer materials as a sensing layer in environmental monitoring has also been developed. Thus, the incorporation of biopolymer and conducting polymer materials with various methods has shown promising potential with sensitively and selectively toward heavy metal ions. In this feature paper, selected recent and updated investigations are reviewed on biopolymer and conducting polymer-based materials in sensors aimed at the detection of heavy metal ions by optical methods. This review intends to provide sufficient evidence of the potential of polymer-based materials as sensing layers, and future outlooks are considered in developing surface plasmon resonance as an excellent and valid sensor for heavy metal ion detection.

## 1. Introduction

Living things such as animals, humans and plants required some basic needs such as air, food and water that are clean and adequate for continue survive. However, due to the growth of industrialization and urbanization, environmental sources, especially of water, have been polluted and this is predicted to become worse over time. This global issue is caused by agricultural and industrial waste products that are disposed into the oceans and rivers. Thus, the water is contaminated by organic and inorganic pollutants, toxic heavy metals, metalloids and synthetic organic chemicals.

Heavy metal ions have become one of the major water pollutants, known as a toxic and non-biodegradable substances that cause serious issues for the environment and human health [1]. For instance, Cd^2+^, Hg^2+^ and Pb^2+^ are dangerous as they can exert impacts on the human body resulting in several fatal diseases such as kidney dysfunction, brain cancer and metabolic disorders [2,3,4]. The contaminant levels of heavy metal ions in drinking water should follow the recommendations from environmental agencies such as the World Health Organization (WHO), the US Environmental Protection Agency (EPA) and the European Medical Agency (EMA) [5]. However, the concentrations of trace heavy metal ions have been exceeded and do not meet the allowed range. Therefore, the development of heavy metal ion sensors for the detection of pollution in water resources and the environment has been extensively developed by researchers worldwide.

To determine heavy metal ions in aqueous solutions effectively, various types of methods have emerged. The conventional analytic methods that are commonly used include atomic absorption spectroscopy (AAS), inductively coupled plasma–mass spectrometry (ICPMS), anodic stripping voltammetry (ASV) and X-ray fluorescence spectroscopy (XRF) [6]; these have successfully detected heavy metal ions in low concentrations and showed good selectivity to analytes. Unfortunately, their implementation is hampered by some limitations, such as the requirement of complex operation and the need for expensive instruments; these methods are also time-consuming processes. In light of these limitations, researchers have been attentive to optical methods for sensing a variety of heavy metal ions and other target materials, as electrochemical, electronic analyses and other modern methods [7] do not offer the same features as optical sensors, which are facile, rapid, cost-effective and have excellent sensitivity and selectivity towards analytes.

**Figure 1:**
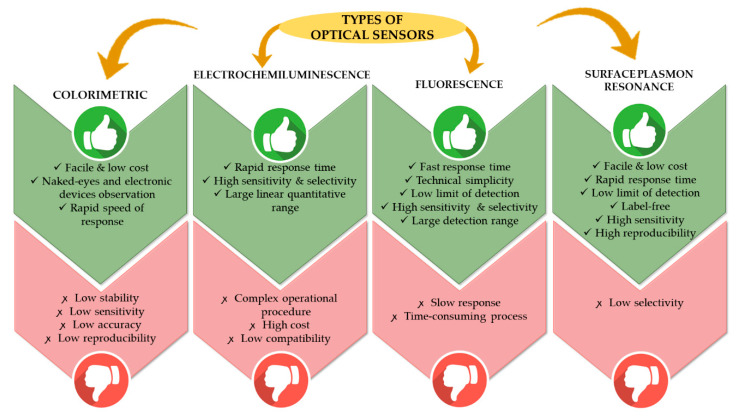
Comparison of advantages and disadvantages of optical sensors for heavy metal ion detection.

Thus, research focused on the technology of optical sensors is promising in detecting heavy metal ions. Colorimetric, electrochemiluminescence, fluorescence and surface plasmon resonance are the list of optical sensors that have been developed to overcome the limitation of the before-mentioned sensors. A colorimetric sensor is an optical sensor involving changes of color of an indicator upon interaction with the analyte, which can be observed easily by naked eye or electronic devices. However, this technique has low sensitivity and low accuracy in producing a result [8]. Next, fluorescence is the optical phenomenon of light emission of certain molecules after the absorption of photons. This technique has a limitation in that it has a long response time. Electrochemiluminescence involves the formation of electrochemically generated species that interact and undergo electron transfer reactions, which consequently emit light from excited states [9]. This technique needs rather complicated operating processes. Surface plasmon resonance is an optical process of the interaction between light and metal-dielectric materials [10]. This optical method also has its own advantages and disadvantages and different sensing abilities in terms of selectivity and sensitivity. Figure 1 summarizes and compares the advantages and disadvantages of optical sensors for heavy metal ion detection.

Therefore, the effectiveness and efficiency of sensors should be improvised, and this has led to numerous works on the fabrication of sensing layers, which rely on different types of materials that have been used. Over the years, different materials such as graphene oxide [11,12,13], polymers [14,15,16] and quantum dots [17,18,19] have been incorporated with optical sensors to enhance the sensitivity and selectivity. Novel and unique properties of materials are considered to determine suitable sensing materials. Because of interesting naturally-based characteristics, biopolymers including cellulose, nanocrystalline cellulose and chitosan have been actively investigated in the last decades. Intensive research works have been devoted to the preparation and characterization of biopolymer materials and applied as environmental sensors. Cellulose is one of the most common polysaccharides and also an unlimited organic material in the Earth. This colorless and odorless polymer consists of several hundred to ten thousand linear chains of β-1,4 linked to d-glucose units with the formula of (C_6_H_10_O_5_)_n_ [20]. The non-toxic polymer also possesses many interesting and promising properties, including biocompatibility, high adsorption capacity, hydrophilicity, relative thermostability and changeable appearance [21]. Meanwhile, nanocrystalline cellulose is a cellulose nanocrystal with nanoscale diameters of 1 to 5 nm and lengths in the range of 150 to 300 nm. By an acid hydrolysis process, this polymer can be synthesized, and the properties of the material can be enhanced for wide application [22].

Next, chitosan is a linear amino polysaccharide of glucosamine and an *N*-acetyl glucosamine unit that can be obtained by alkaline deacetylation of chitin. This polymer can be easily synthesized by surrounding resources, abundantly available from the shells of crab, prawn, shrimps, fish scale and also from plant-based material [23]. It is an excellent stabilizer of metal nanoparticles, has good biocompatibility and is a low cytotoxicity material. With hydrophilic properties and a large number of amino and hydroxyl groups, the polymer is also able to undergo several chemical modifications; hence, it governs the properties of the material [24]. Owing to many natural promising characteristics, the above-mentioned materials have received much attention due to their own unique and novel properties. Thus, biopolymers are an excellent matrix for sensing applications as a promising material in developing a selective and sensitive sensor towards the targeted analytes, particularly heavy metal ions.

**Figure 2:**
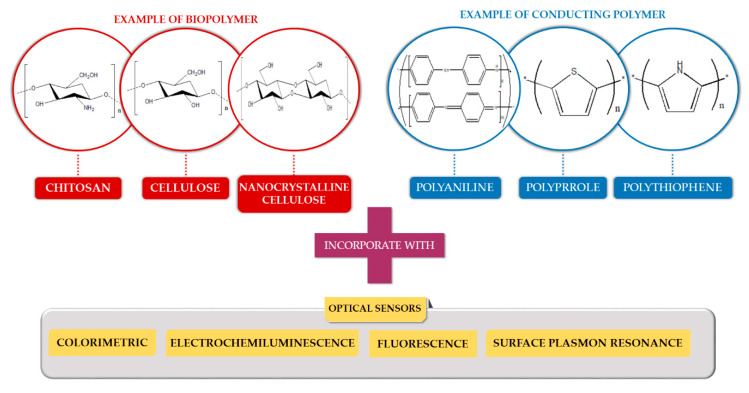
Illustration of biopolymers and conducting polymers incorporated with various types of optical sensors.

Apart from that, conducting polymers such as polyaniline, polypyrrole and polythiophene are also promising materials in specific applications. Their electronic conducting, optical and chemical properties have received great attention, due to their unique characteristics such as electrical conductivity, good environmental stability and non-toxicity [25]. These unique polymers can be considered in interdisciplinary science and technology, particularly in actuators, light-emitting diodes (LED), transistors, supercapacitors and optical sensors [26]. Therefore, conducting polymers have also showed impressive performance and potential to be incorporated with several optical techniques in fabricating effective heavy metal ion sensors. Figure 2 illustrates the biopolymers and conducting polymers that have been incorporated with optical sensors to detect heavy metal ions.

Henceforth, this paper firstly reviews the incorporation of optical sensors involving colorimetric, electrochemiluminescence and fluorescence with biopolymers and conducting polymers. Then, the paper provides an overview of the surface plasmon resonance technique in the fabrication of heavy metal ion sensors.

## 2. Application of Polymer Based Material with Optical Sensor for Heavy Metal Ion Detection

### 2.1. Biopolymer-Based Material

As reported, the metal-chelating ability of chitosan can be enhanced by chemical modification of the amino group. Thus, in 1992, Kurauchi et al. originated the earliest work of biopolymer material using chitosan modified with 5-formyl-3-hydroxy-4-hydroxymethyl-2-methylpyridine (FHMP) and immobilized it on an agarose gel to act as a fluorogenic probe. The sensor showed a higher sensitivity to Zn^2+^ in a range of 0 to 25 µM with a detection limit of 1 µM. They believed that during chelate formation, the participation of the hydroxyl groups adjacent to the amino groups contributed to the strong fluorescence of Zn^2+^ chelates of pyridoxal/amino sugar Schiff bases. The responses to Cd^2+^ and Ga^3+^ were lower than Zn^2+^ by about 1 mM [27].

After a decade, Lai et al. fabricated a fluorescence probe using cadmium sulfide quantum dots (CdSQDs) modified with chitosan for the determination of Cu^2+^. Other than high chelating ability with metal ions, the amino and hydroxyl groups of chitosan were also good capping groups and could disable the agglomeration of quantum dots during growth. They believed that the fluorescence of quantum dots (QDs) was quenched efficiently and resulted in a linear response between fluorescence intensity and Cu^2+^ concentration within the wide range of 8 nM to 3 µM. Thus, the fluorescence probe had remarkably high sensitivity with a limit of detection of 1.2 nM [28].

Later, in 2012, Wang et al. synthesized a fluorescence chitosan chemosensor through a substitution reaction of amino groups of chitosan nanoparticle (CSN) with dansyl chloride (DA). According to this project, the fluorophores were covalently bonded onto the chitosan nanoparticles network, which was attributed to the higher fluorescence intensity of DA-CSN. They reported that the intensity of fluorescence decreased when the Hg^2+^ concentration increased up to 1 mM with a detection limit of 1 µM [29].

Then, magnetic and fluorescent bifunctional chitosan nanoparticles (MF-CSNPs) were prepared via electrostatic interaction by Liu et al. [30]. By surface modification of the chitosan layer with Fe_3_O_4_–chitosan magnetic nanoparticles and cadmium selenide quantum dots, the stability of MF-CNPSs was improved and the fluorescence probe was further used to detect Cu^2+^. The measured detection limit was 0.724 µM with a good linear relationship observed over 1.967 to 393.416 µM of Cu^2+^ concentration.

In 2014, Dang et al. developed a label-free and sensitive electrogenerated chemiluminescence (ECL) aptasensing scheme using a chitosan/Ru(bpy)_3_^2+^/silica (CSRuS) nanoparticle-modified glass carbon electrode. This ECL aptasensing method was used to detect different concentrations of K^+^ in a range of 0 to 9 nM, which resulted in a limit of detection of 0.3 nM. The proposed ECL sensing mechanism could sensitively discriminate the free state of G-rich aptamer from its complex state and it was satisfactory for other applications such as protein detection [31].

A sensor of three-dimensional chitosan hydrogel with superior fluorescence properties for Hg^2+^ detection was successfully fabricated by Geng et al. in 2015. This sensor was prepared by the crosslinking approach of modifying chitosan fibers with glutaric dialdehyde (GD). They stated that the fluorescence quenching was caused by the formation of a new complex by an interaction of Hg^2+^ with GD fluorophores. Furthermore, the three-dimensional fluorescent chitosan hydrogel may undergo the oxidation of fluorophores or reduction of Hg^2+^. The sensor can detect Hg^2+^ as low as 0.9 nM with a range of up to 50 nM [32].

Another study of Hg^2+^ detection in 2015 by Chen et al. was also done. However, they used colorimetric detection and chitosan-functionalized gold nanoparticles (AuNPs) to play a role as a signaling probe. In this study, a decrease in absorbance peak with a color change from red to blue was observed. A linear range of 0.05 to 9 µM with a detection limit of 1.35 µM was also obtained [33].

At the same time, Chauhan et al. produced an innovative procedure for the fabrication of a facile colorimeter sensor using a new chitosan thiomer to detect Hg^2+^. The sensor probe was synthesized through microwave irradiation of a chitosan isothiouronium salt intermediate and low coat thiourea reagent. As the concentration increased from 0 to 498.5 µM, the color of the sensing polymer solution was gradually changed from colorless to yellow and brown, which resulted in the calculated limit of detection at 2.318 µM [34].

Meanwhile, Shi et al. used magnetic core–shell chitosan microspheres modified with a rhodamine spirolactam (Rho-MCS) as fluorometric probes for Hg^2+^ detection. The fluorescence intensity was observed as the Hg^2+^ concentration changed in a range of 0.5 to 7 µM, and with an established limit of detection of 0.015 µM [35]. Meanwhile, Shi et al. prepared a rhodamine-based fluorescent probe onto the surface of chitosan as a sensor and adsorbent to recognize and remove Hg^2+^ from water. It was clearly seen that the fluorescent intensity increased with the enhancement of Hg^2+^ concentration up to 6 µM. The results also displayed a distinguishable color change from pale yellow to pink and the ability to achieve a limit of detection of 3.42 µM [36].

Still, in 2015, Mehta and co-workers developed a colorimeter sensor for sensing Cd^2+^ based on chitosan dithiocarbamate functionalized gold nanoparticles (CSDTC–AuNPs). The probes produced a color change from red to purple/blue, which indicated a higher degree of CSDTC–AuNP aggregation as the concentration of Cd^2+^ increased. The sensor displayed good selectivity and sensitivity with a limit of detection of 0.063 µM for detecting Cd^2+^ in water samples [37].

A colorimeter sensor for detecting Hg^2+^ was designed by Nivethaa et al. using a chitosan–silver polymer matrix nanocomposite. There were several probes prepared containing different weight percentages of silver; however, the probe with 50 weight percent of silver was further used in detecting Hg^2+^. In a range of 0 to 500.870 µM, the detection limit of 7.2 nM was calculated [38].

In 2016, another fluorescence-based sensor for Cu^2+^ sensing containing carbon dots on carboxymethyl chitosan-wrapped Fe_3_O_4_ nanoparticles was designed by Kumar et al. [39]. The sensing performance of the fluorescent magnetic nanoparticles was evaluated by the quenching efficiency of carbon dot-attached magnetic nanoparticles that decreased when the concentration of Cu^2+^ increased. From that, the excellent reliability of this sensor was confirmed with the detection limit of 0.56 µM.

The following year, Nivethaa and co-workers improved the detection of Hg^2+^ by a synthesized chitosan/silver–gold (CS/Ag–Au) nanocomposite via the chemical reduction method. A plot of absorbance intensity against Hg^2+^ concentration exhibited a proportional relationship and found that the calculated limit of detection was better than the previous study with a value of 0.5 nM. This nanocomposite also showed its selectivity only towards Hg^2+^ compared to other metal ions [40].

In 2017, Tian et al. developed a low-cost colorimetric method in sensing Hg^2+^ involving chitosan stabilized gold nanoparticles and 2,6-pyridinedicarboxylic acid. Interestingly, this method, based on the induced aggregation of nanoparticles, offered many advantages for on-site analysis. Upon Hg^2+^ concentration increase, the original color of the solution changed from red to blue. This sensor showed an excellent selectivity towards Hg^2+^ among several metal ions and exhibited linearity in a range of 0.3 to 5 µM [41].

Wang and Gao synthesized a fluorescent probe based on chitosan/silver nanocomposite films using NaBH_4_ as a reductant of AgNO_3_ for the linear detection of Al^3+^ between 2 and 180 µM. Chitosan acts as a stabilizer due to it being a strong chelating agent for metals. After at least a fivefold higher sensing capability, it could be concluded that this probe had higher repeatability and was highly sensitive to the presence of Al^3+^ [42].

Later, tetraphenylethylene (TPE), also known as aggregation-induced emission (AIE) active material, was linked to chitosan chains for monitoring Cu^2+^. Liu et al. observed a proportional relationship between quenched fluorescent intensity and concentration of the Cu^2+^ range up to 100 µM. TPE–chitosan has a higher water-solubility and absorbability due to properties of chitosan that stabilize complexation with several heavy metals [43].

The same as previous reports, detection of Cu^2+^ based on the fluorescent property of fluorescein isothiocyanate (FTIC)-labeled chitosan (CS) oligomer was reported by Lee et al. They used distilled water as a medium to mix 100 mL of FTIC in EtOH and 10 mL solution of chitosan oligomer, and the mixture was then tested with several metal ion solutions. As a result, CS–FTIC was shown to have a higher selectivity towards Cu^2+^ with a limit of detection of 60 µM. This resulted from energy transfer between FTIC and Cu^2+^, which led to the change of fluorescence quenching [44].

In 2018, a study on the optical detection of Hg^2+^ based on an enhancement of the peroxidase-like activity of chitosan–gold nanoparticles (CS–AuNPs) was done by Jiang et al. [45]. Based on colorimetric images, obvious changes were observed from a blue solution transformed to a deeper color upon addition of 0.04 µM Hg^2+^. This confirmed that peroxidase-like activity produced a characteristic color due to the oxidation of chromogenic substrates. Two main factors of enhanced catalytic ability were achieved by the aggregation of CS–AuNPs induced by Hg^2+^. Next, there was an attraction of nanoparticles to 3,3′,5,5′-tetramethylbenzidine during Hg^2+^ detection. The calculated limit of detection was equal to 0.02 µM. Thus, this colorimetric sensor had huge potential for application in real samples.

A colorimetric paper stripe was prepared by incorporation of silver-doped cadmium selenide quantum dots (CdSAg QDs) and chitosan-coated cellulose for sensing Hg^2+^. Butwong et al. reported that a remarkable color change from yellow to deep brown was due to Hg^2+^ being trapped by mercaptoacetic acid on CdSAg QDs. Other than that, a proportional relationship between color intensity and the concentration of Hg^2+^ was observed; thus, a visualized detection limit of 124 µM was also obtained. Thus, an efficient mercury test stripe was successfully fabricated with high sensitivity and repeatability for on-site sensing [46].

Sharma et al. synthesized a sensitive and selective Hg^2+^ sensor by interacting with the amine group of chitosan and a carboxylic acid group of 3-mercaptoppropanoic via amide coupling. The colorimetric sensor of thiol terminated chitosan capped silver nanoparticles (Mod-CS–AgNPs) resulted in a fast color change from yellow to colorless within 5 s upon the addition of Hg^2+^. The peak intensity was decreased with the increasing concentration of Hg^2+^ within a range up to 0.4 µM, and the limit of detection for Hg^2+^ was calculated to be 0.017 µM. As reported before, the redox reaction between silver nanoparticles and Hg^2+^ caused silver nanoparticles to be deformed, which was confirmed by a blue-shift in the UV–Vis spectra [47].

Next, Amanulla et al. analyzed carbon-based material-decorated nanoparticles colorimetrically for determination of Hg^2+^. The composite also included chitosan as a reducing and stabilizing agent to enhance the selectivity and sensitivity of the sensor. From their observation, the Au@S-g-C_3_N_4_ composite proved the recognition capability of Hg^2+^ even in the presence of several metal ions. The excellent specificity was attributed to the strong binding affinity with heteroatoms of S-g-C_3_N_4_ and also an amalgam formed by the strong binding energies of gold–Hg^2+^ interactions. The linear plot of absorption spectra against the different concentrations of Hg^2+^ was obtained, and it signified that the limit of detection was approximately 0.275 nM [48].

In 2019, Hu et al. reported another chitosan-based composite that was prepared as functionalized paper strips by immersing a common filter paper into chitosan–gold nanoparticles overnight. Next, the ready paper strip was immersed in the Hg^2+^ solution under ambient conditions. After 5 min, a color change from dark-red to yellow was recorded, and a detection limit of 0.05 µM was visualized by naked eye. Another limit of detection was also obtained by spectral analysis, i.e., 3.2 nM. However, both values were still in the range defined by the World Health Organization (WHO) [49].

Another green approach in preparing gold nanoparticles with chitosan (CS–AuNPs) was done by Zhao et al. The use of chitosan has been discovered in wide areas as it has excellent properties and can act as reductant and stabilizer in aqueous solution. Firstly, Ag^+^, as the targeted metal ion, was interacted with a gold core to enhance the metallic bond; then it was reduced and deposited on the surface of AuNPs. The proposed composite showed a distinguishable color change from pink to orange when the concentration of Ag^+^ increased in the range of 1 to 100 µM. With a detection limit of 0.13 µM, it was shown that this method has excellent sensing properties and can be applied in the future [50].

Next in 2007, the earliest study of optical sensors with cellulose was done by Egorov et al. [51]. They designed a colorimetric test using the ionic liquid 1-butyl-3-ethylimidazolium chloride and 1-(2-pyridylazo)-2-napthol to determine Zn^2+^, Mn^2+^ and Ni^2+^. Cellulose dissolution in ionic liquids and immobilization of organic reagents on a cellulose medium was the motivation of this study; 1-(2-pyridlazo)-2-naphtol (PAN) is an organic reagent that has been immobilized on cellulose film to undergo colorimetric measurement for the above-mentioned metal ions. As a result, PAN-modified cellulose has a higher detection limit compared to other immobilized organic reagents. This might be due to the concentration of the reagent in composite due to low color contrast. Additionally, this cellulose film was reusable and can be used as a quantitative determination of transition metal cations.

A study on functional surface modification of natural cellulose by Zhang and Huang via immobilization of ruthenium dye N719 monolayer onto titania ultrathin gel film pre-coated cellulose nanofibers was discussed. The cellulose-based composite of 15-layer titania film and N719 monolayer ((TiO_2_)_15_/N719) modified filter paper was treated with different concentrations of Hg^2+^ in a range of 50 nM to 100 µM. The selectivity test on Hg^2+^ was done by exposing ((TiO_2_)_15_/N719) modified filter paper with a mixed aqueous solution consisting of Cu^2+^, Mg^2+^, Pb^2+^ or Zn^2+^. It revealed the value of a detection limit was approximately 0.049 µM and it indicated that this sensor has high sensitivity and selectivity towards Hg^2+^ over other metal ions [52].

There was an innovative study that was conducted on a multifunctional sensor based on electrospun fiber membranes for sensing metal ions. In 2011, Wang and coworkers developed a multifunctional fluorescence probe by encapsulating 1,4-dihydroxyanthraquione solution onto cellulose nanofiber films. The detection of Cu^2+^ and Cr^3+^ was based on the fluorescence emission changes of different probes. Firstly, 1,4-dihydroxyanthraquione solution doped cellulose (1,4-DHAQ@CL) was immersed in a varied concentration of Cu^2+^, and the fluorescence ratio showed a linear dynamic detection range of 2.5 nM to 37.5 nM. Next, the determination of Cr^3+^ involved another nanofiber film codoped with Cu^2+^, which was (1,4-DHAQ)-Cu@CL. For this time, a linearity relationship was observed ranging from 2.5 nM to 25 nM. Thus, this multifunctional sensor has potential as an effective fluorescence probe for sensing other heavy metal ions [53].

A sensitive fluorescence probe towards Fe^3+^ using pyrene bearing cellulose nanocrystals (Py–CNC) was prepared by a three-step method. Hydrophobicity is the limiting factor of many Fe^3+^ selective fluorescence probes. Thus, to overcome this problem, cellulose nanocrystals were used to increase the solubility of Py–CNC in water. When Fe^3+^ concentration was raised to 5 mM, the difference in fluorescence emission was observed and resulted in a limit of detection of 1 µM. The electron–energy transfer process in exciting pyrene fluorophore and Fe^3+^ caused quenching to occur, which enabled a non-radiative deactivation pathway to happen [54].

In 2013, Li et al. demonstrated a simultaneous detection of Pb^2+^ using a novel solid-phase nanofibrous material consisting of deacetylated cellulose acetate membrane with pyromellitic dianhydride (DCA–PMDA). Firstly, they synthesized the cellulose acetate nanofibrous material (CA NFM) through the electrospinning before further use in colorimetric detection. The interaction between DA–PMDA and Pb^2+^ was attributed to the presence of –COOH groups in forming the Pb^2+^–carboxyl coordination. As the color changed from white to dark yellow–brown, a low naked-eye detection limit of 0.048 µM was obtained [55].

You et al. discovered a novel cellulose polyampholyte applied in a label-free colorimetric sensor for recognition of cysteine and Hg^2+^. The polymeric system consisted of carboxylethyl quaternized cellulose (CEQC) that played the role of a stabilizer for gold nanoparticles (AuNPs). Initially, they measured different concentrations of cysteine based on the UV–Vis mechanism by the Au–CEQC solution. Then, detection of Hg^2+^ using another Au–CEQC solution was done where cysteine was added to induce the aggregates of AuNPs. As could be seen, the color solution changed from red to purple and later turned to blue. The sensing layer indicated the value of the detection limit at 0.04 µM for the prepared Hg^2+^ solution. Meanwhile, for real sample application, the value of the detection limit decreased to 0.03 µM [56].

A year later, another three-step procedure by Xu et al. was done to design rhodamine derivative-modified cellulose filter paper as a sensor for Hg^2+^ ions. The filter paper with surface modified spiorolactam rhodamine derivatives (CL-g-RD) was prepared by immobilization of alkyl bromide initiators on cellulose filter papers, which was then followed by surface initiated atom transfer radical polymerization and lastly thru post-functionalization of the reactive surface in ester-amine solution. The detection limit of the solid-state sensor was about 50 µM, and a color change from colorless to pink happened when the Hg^2+^ increased, ranging from 0 to 10 mM. They also found that CL-g-RD could be applied as a disposable solid-state sensor with a fluorescence recovery rate of about 85% [57].

Schiff bases acquired by the reaction of aldehyde and amine are one of the main components in the fluorescent analysis of metal ions. Thus, Kumari et al. investigated the sensitivity and selectivity of a cellulose–lysine Schiff base (C_10_–O–Cell–HC≡N–Lys) for Hg^2+^ absorption. According to this study, the presence of hydrophobic alkyl groups attached to a –C≡N– bond would improve the stability and reusability of the sensor. There was formation of a colored complex between the Schiff base with metal ions when the initial color of Hg^2+^ was turned to yellow, and the lowest visual detection limit was obtained at 49.853 µM [58].

**Figure 3:**
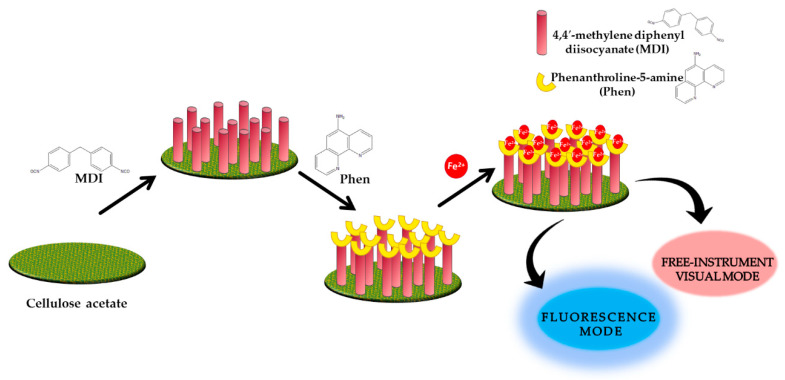
Detection strategy of Fe^2+^ by Phen-MDI-cellulose based on multimode sensors [59].

Three years later, Nawaz et al. invented the cellulose-based sensor (Phen-MDI-CL) containing 1,10-phenanthroline-5-amine (Phen) and using 4,4′-methylene diphenyl diisocyanate (MDI) as a cross-linker, as shown in Figure 3. By fluorescence measurement, there were changes in emission intensity of Phen-MDI-CL as the Fe^2+^ aqueous solution was added, the detection limit of which was obtained at 46.6 nM. Meanwhile, by instrument-free visual mode, the detection limit was 895 nM. Consequently, Phen-MDI-CL can be implied as a multimode sensor after showing a rapid response and visible sensing of Fe^2+^ ions [59].

Another cellulose-based carbon nanoparticles composite was prepared by Yun-Fei et al. as a fluorometric assay for sensing Pb^2+^. The carbon nanoparticles were obtained by treating nanocrystalline cellulose (NCC) with chlorine and thionyl chloride, subsequent to dehydrating carbonization, oxidation and modification by ethylenediamine (EDA). With the increasing concentration of the analyte within a range of 18.7 nM to 0.5 µM, there was a significant decrease in intensity due to fluorescence quenching. Then, the lowest detection limit of 0.024 µM was obtained for NCC–EDA as a fluorescent probe to detect lead ions [60].

The next year, another promising biopolymer was synthesized by Milindanuth et al. using bacterial cellulose (BC) with rhodamine B derivative (Rh-2). They believed that by using an ultra-fine network of cellulose nanofibers as a sensor, it could enhance the accessibility to metal ions. The BC paper-based sensor was treated by immersing it in Rh-2, and they proceeded to use it in the colorimetric mechanism. A visible change of solution could be seen after it turned to pink as soon as Cu^2+^ was added. The linear relationship between the absorbance and the intensities against the concentrations of copper ions in a range of 4–128 µM was observed, with values of linear correlation R = 0.9993 and R = 0.991 [61].

Recently, Faham et al. discovered the potential of curcumin embedded bacterial cellulose (CEBC) as a nanopaper-based analytical device (NAD) for monitoring levels of Fe^3+^ concentrations in a colorimetric system. The preparation of NAD/CEBC involved laser printing technology to obtain the hydrophilic and hydrophobic areas on bacterial cellulose nanopaper. From their observations, the color was changed to dark yellow as the UV–Vis peaks decreased at λ = 427 nm. Due to the formation of a Fe^3+^–curcumin complex, the absorbance and color intensity of the colorimetric mechanism was decreased. This output signal was monitored by a smartphone camera and spectrophotometer, which resulted in detection limits at 7.8 nM and 8.2 nM, respectively [62].

### 2.2. Conducting Polymer-Based Material

Owing to the excellent properties of conducting polymers, these polymers are also options for developing optical sensors for heavy metal ion detection. In 2011, Ding et al. used a different method in preparing a colorimetric probe, which is an electro-spinning/netting (ESN) method. This facile process produced a homogenous structure of polyaniline/polyamide-6 (PANI/PA-6) nano-fiber/net (NFN) membranes, and it gave an effect to colorimetric sensing properties in terms of purity and homogeneity of color. This sensor strips displayed responses towards several concentrations of Cu^2+^ with the lowest detection limit at 15.737 µM. As the concentration of analytes increased from 0 to 1.573 mM, the solution changed from white to dark blue. They also stated that this sensor was possible to be used as a pH sensor as the solution color changed when pH was increasing [63].

**Figure 4:**
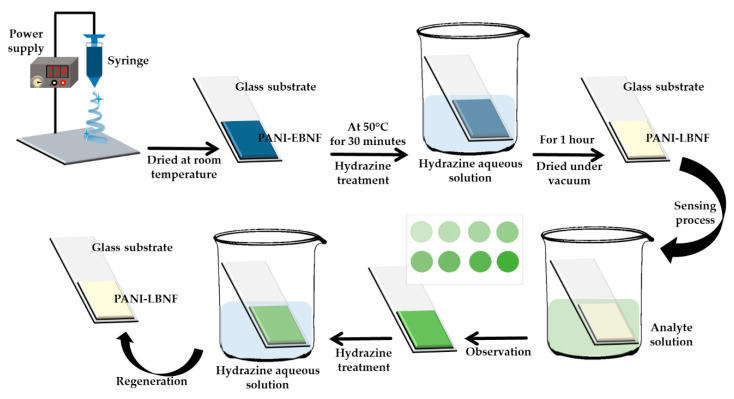
Schematic representation of preparation on fully reduced polyaniline (PANI-LB) sensing membranes [64].

In 2014, Si et al. introduced a homogenous and hierarchical nanofibrous structure with better colorimetric sensing properties by combining fully reduced polyaniline (PANI-LB) and an electrospinning nanofabrication technique (Figure 4). The prepared fiber probe was evaluated with a variety of cation analytes, and there were no obvious reflectance spectra changes except for Hg^2+^; at 440 and 645 nm, the reflectance intensity seemed to be decreased. The PANI-LB nanofibrous sensors not only possessed high sensitive colorimetric responses with a low detection limit of 5 nM, but also high reversibility for a label-free Hg^2+^ sensor [64].

Later, a study on the fluorescence sensor for recognition of Hg^2+^ using polyaniline/carbon dot (PANI/CD) nanocomposites was further developed. Wang et al. reported that the relationship between fluorescent intensity and concentration of Hg^2+^ exhibits linearity in the range of 0.05 to 1 µM and the lowest detection as low as 0.8 nM. By fluorescence resonance energy transfer, the fluorescence quenching between CD and PANI was confirmed. Furthermore, the high selectivity of PANI/CD towards Hg^2+^ was suggested by the rapid chelating process and a strong thermodynamic affinity of polyaniline [65].

In preparing conjugated polymer, there are a few limitations, such as low yield, low stability and the requirement of complex conditions. Therefore, Wang et al. proposed the preparation of fluorescent polyaniline (PANI) microspheres functionalized on the surface of silver nanoparticles under mild conditions involving the simply controlled oxidation of aniline. This PANI-based fluorescent probe possessed a high sensitivity to Hg^2+^ with the lowest detection limit at 0.86 nM. The polarization and deformation through the interaction of Hg^2+^ and nitrogen atoms of PANI efficiently occurred and was caused by a larger ionic radius of Hg^2+^ compared to other metal ions. Thus, this study successfully prepared a convenient Hg^2+^ probe for future environmental monitoring [66].

Water pollution commonly happens due to the contamination of toxic metal ions. However, arsenic contamination will bring the worst effects to humans as well as flora and fauna because it is easy to find in environmental resources. Thus, Saikia and Karak were motivated to develop an efficient fluorimetric As^3+^ sensor using a polyaniline nanofiber/carbon dot (PANI/CD) nanohybrid. A linear response between As^3+^ concentration and fluorescence quenching was obtained, and they managed to achieve a low limit of detection of 1.3 pM. The PANI/CD was selective towards As^3+^ due to the formation of the complexation and electrostatic interaction during the experiment [67].

In 2016, a fluorescence sensor prepared by Tavoli et al. with a new anionic dopant, Tiron, was used with nanostructured polypyrrole film for electrically controlled sensing systems of Fe^3+^. The polypyrrole–Tiron (PPy–Tiron) was coated on a transparent electrode via electrodeposition, which enhanced the sensing behavior to be observed. By applying −0.5 V potential to the PPy–Tiron film, the fluorescence intensity was quenched upon the addition of Fe^3+^, and the color changes of the film were observed. The empty d shells and efficient quench of the fluorescence intensity through energy or electron transfer were the factors leading to the selectivity of PPy–Tiron film regarding Fe^3+^. In a concentration range of 0.05–1 µM, the calculated limit of detection was 0.01 µM [68].

Recently, a novel benzene sulfonic acid doped polypyrrole (PPy–BSA) thin film as a fluorescent sensor for the determination of Cu^2+^ and Pb^2+^ was synthesized by Lo et al. Firstly, PPy–BSA was prepared electrochemically by cyclic voltammetry on an indium tin oxide (ITO) electrode. The fluorescence probe showed strong fluorescence quenching due to the binding interaction of the thin film with analytes. The limits of detection were 3.1 nM and 0.018 µM for Cu^2+^ and Pb^2+^, respectively; it was obtained from a linear Stren–Volmer relationship in the range of 0–9 µM [69]. Table 1 summarizes the findings of different polymers incorporated with various optical sensors for metal ion detection.

## 3. Surface Plasmon Resonance

With beneficial features, i.e., cost-effectiveness, high sensitivity and simple procedure required, the surface plasmon resonance (SPR) sensor has become one of the complementary optical techniques with good capability in biological and environmental analysis. The basic principle of this surface-sensitive technique, by observing the changes of the resonance angle by adding the interface between media and a metal thin film, resulted in the change in the refractive index of the metal surface.

A phenomenon called surface plasmon happens when an electromagnetic wave of polarized monochromatic light hits a metal thin film, causing collective oscillation of free electrons on the surface metal–dielectric interface. The incident light is absorbed and the electrons at the interface receive the energy. Thus, at a specific incident angle, the momentum of the surface plasmon is equivalent to the momentum of the incident photon. At this moment, resonance will occur, and the intensity of reflected light will be reduced. As a consequence, a sharp shadow called surface plasmon resonance (SPR) is observed.

**Figure 5:**
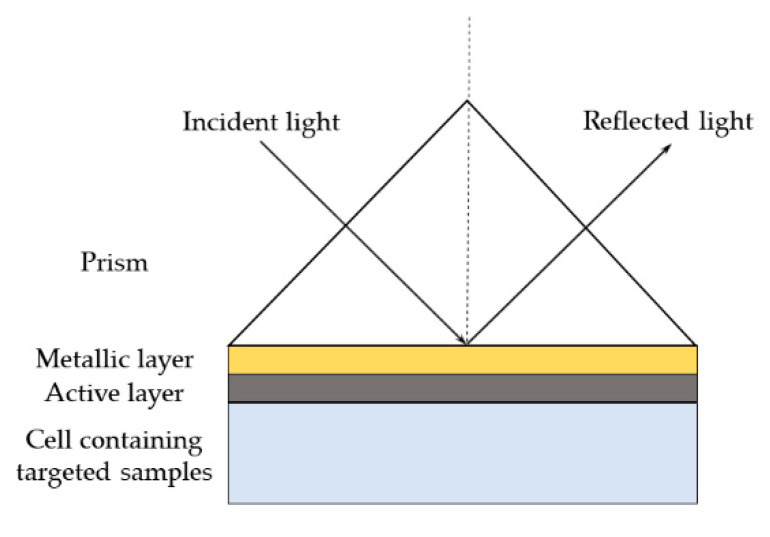
Schematics of the Kretschmann configuration of surface plasmon resonance prism.

There are two configurations of a prism coupler, which are the Kretschmann configuration and the Otto configuration. The prism coupler is required in the SPR sensor to ensure that the excitation of the surface plasmon can generate in two semi-infinite media. The most common setup, the Kretschmann configuration, is shown in Figure 5. A metal (usually gold or silver) thin film is placed directly onto the horizontal surface of the prism with no air gap, as the presence of an air gap will decrease the SPR efficiency. Thus, this configuration is more practical in SPR measurement.

### 3.1. Incorporation of Biopolymer with Surface Plasmon Resonance

**Figure 6:**
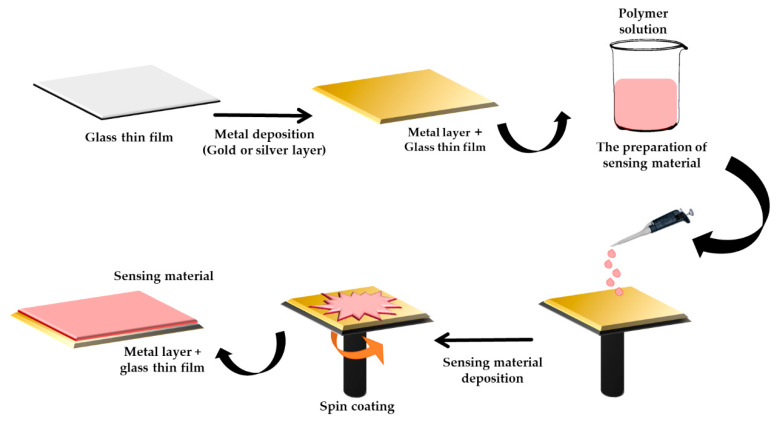
Schematic of the fabrication process of a thin film for surface plasmon resonance [12].

As a consequence, many studies have been done by researchers to fabricate the active layer to incorporate with the SPR sensor. As can be seen in Figure 6, the fabrication of the active layer starts with the deposition of the metal layer and is followed by the deposition of sensing material. Therefore, the sensitivity of the sensing layer can be determined, and the detection limit of the sensor also can be obtained according to the response of the SPR curve towards the concentration of the target metal ions. Thus in 2008, an introductory work was done by Mcllwee et al. [70], where they reported the formation of homogenous thin chitosan films on the SPR interface. The homogenous thin film achieved optimal conditions by spin-coating a solution at 5000 rpm. The best SPR signal was obtained when the chitosan had the thinnest layer of 10 nm. As a result, the specific binding of Fe^3+^ ions with thin chitosan film as low as 4.477 µM and up to 1.79 mM was determined.

The following year, Fahnestock et al. used a gold nanoparticle (AuNP)/chitosan composite film in localized surface plasmon resonance to study the selective removal of hexachromium ions. They reported that at pH 6.8 in deionized water, CS/AuNP was able to selectively detect Cr^6+^ ions with a detection limit of 192.322 µM. Besides, the presence of Na^+^ influenced quantitative Cr^6+^ detection compared to Cr^3+^ [71]. On the other hand, Fen et al. in 2011 detected mercury and copper ions by chitosan cross-linked glutaraldehyde solution. The relationship of resonance angle and the concentration of heavy metal ions in solution is directly proportional, and they observed that the sensing layer was more sensitive to Hg^2+^ than Cu^2+^ with detection limits of the sensor as low as 2.493 µM [72]. Still, in 2011, Fen et al. once again studied the potential of SPR in sensing Zn^2+^, Cu^2+^ and Mn^2+^ with the same layer, namely chitosan cross-linked glutaraldehyde. At this time, they reported that the interaction of these metals ions resulted in sensitivity of this sensor layer in the following order: Cu^2+^ > Zn^2+^ > Mn^2+^. The limit of detection for these metal ions was 7.868 µM, 7.76648 µM and 9.101 µM, respectively [73].

In 2012, Fen et al. conducted an SPR sensor for the detection of Pb^2+^. Different from the previous study, they used *p*-*tert*-butylcalix[4]arene-tetrakis (BCAT)-immobilized chitosan thin film. The BCAT-immobilized chitosan enhanced the thin film for adsorption of Pb^2+^ and gave effect to the shift of SPR signals. Concentrations ranging from 0.144 to 24.131 µM could be quantified. Thus, the change in resonance angle is directly proportional to the increase concentration of Pb^2+^. Furthermore, Pb^2+^ is preferentially adsorbed by the BCAT-immobilized chitosan, so that it can be differentiated from Cu^2+^, Hg^2+^, Zn^2+^ Pb^2+^ and Mn^2+^ [74].

Another work by Fen et al. on crosslinked chitosan thin film has been used to build a Pb^2+^ ion sensor. First, the thin film was prepared by the homogenous reaction between chitosan in aqueous acetic acid and the crosslinking agent glutaraldehyde. Then, 0.55 mL of the solution was deposited on gold thin film and was spun by spin coating with settings of 6000 rev/min. With the concentration range from 2.431 to 482.625 µM, the sensing layer could detect as low as 2.431 µM. Moreover, by crosslinking chitosan and glutaraldehyde, there was an interaction between the primary amino and aldehyde terminal (imino bound) from crosslinked chitosan and the Pb^2+^ ion [75].

Gold/chitosan/graphene oxide (Au/CS/GO) nanostructured thin films were prepared by Lokman et al. as another metal ion sensor to detect Pb^2+^. In this work, the comparison between gold–chitosan (Au/CS) and Au/CS/GO nanostructured thin films could be observed based on all characterization results, including the SPR response. According to field-emission scanning electron microscopy (FESEM) analysis, rough fractured GO nanosheets were covering the Au/CS thin film, resulting in unevenness and roughness of the Au/CS/GO thin film to enhance the interaction of sensing layer and heavy metal ion molecules. In summary, this work showed that Au/CS/GO was more sensitive to Pb^2+^ compared to Au/CS with a limit of quantification or the lowest concentration of 0.153 µM [76].

In 2015, a study by Fen et al. on chitosan–tetrabutyl thiuram disulfide (CS–TBTDS) as a novel active nanolayer in SPR was carried out. When the concentration of Zn^2+^ increased, there was an increment in the change of resonance angle for Au/CS and Au/CS–TBTDS. However, by comparing the results, there were differences in the detection limit of these two thin films, which were 7.648 µM and 1.530 µM, respectively. In the presence of TBTDS, Au/CS–TBTDS improved the sensitivity from 849.94° M^−1^ to 2092.16° M^−1^. Then, the selective detection of Au/CS–TBTDS was done with other metal ions, namely Zn^2+^, Pb^2+^, Hg^2+^, Cu^2+^ and Mn^2+^. From the results, the sensitivity for this layer followed the sequence of Zn^2+^ > Pb^2+^ > Hg^2+^ > Cu^2+^ > Mn^2+^. They believed that this was due to sulfur donor atoms in the TBTDS ionophore, which had a strong affinity towards Zn^2+^ [77].

The next year, Kamaruddin et al. implemented multi-metallic layers of a gold–silver–gold (Au–Ag–Au) nanostructure with chitosan–graphene oxide (CS–GO) for Pb^2+^ detection by the SPR technique. In their project, tri-metallic layers were fabricated by depositing a 10 nm gold layer on a glass slide, followed by a 40 nm silver layer and another gold layer. Then, by spin coating techniques, CS–GO was deposited on the top tri-metallic layers. From their investigation, the detection limit for Pb^2+^ was 0.48 µM within a concentration ranging from 0.483 to 24.131 µM. They reported that the tri-metallic CS–GO SPR sensor offered great repeatability, precision and stability by the low value of relative standard deviation (RSD) of 0.03 to 0.15 [78].

In the following year, Kamaruddin et al. used the same layer (Au/Ag/Au/CS–GO) to develop an SPR sensor. However, the focus this time was on the binding affinity between CS–GO with Pb^2+^ and Hg^2+^ ions. Thus, both metal ion concentrations could be observed, and the sensitivity of Hg^2+^ was lower compared to Pb^2+^, which had a higher sensitivity of 424,760° M^−1^. Based on the Langmuir isotherm model of the SPR angle shift, the calculated binding affinity constant for Hg^2+^ and Pb^2+^ was 4 × 10^5^ M^−1^ and 7 × 10^5^ M^−1^, respectively. They also discussed that greater electronegativity and ionic radii are the factors why the CS–GO sensing layer was more favorable to Pb^2+^ compared to Hg^2^ [79].

Chitosan (CS), graphene oxide (GO) and valinomycin (V) were used to synthesize the ionophore doped graphene-based bionanocomposite solution for the detection of K^+^ by Zainudin et al. [80]. The interaction of the CS–GO–V thin film and deionized water was first carried out. Next, the different concentrations of K^+^ (10–100 M) were injected into a cell and left for 10 min in contact with the thin film. Then, the control resonance angle by deionized water was compared to various concentrations of K^+^. The prepared sensor had a sensitivity value of 370.652° M^−1^, and the detection limit was 0.0256 µM.

In 2018, a 4-(2-pyridylazo) resorcinol–chitosan–graphene oxide (PAR–CS–GO) thin film for the detection of Co^2+^ was reported by Saleviter et al. They mixed 50 mL prepared chitosan solution, 10 mL graphene oxide solution and 5 mL of 1.5 × 10^−3^ g/mL PAR solution before depositing PAR–CS–GO on top of a gold layer by using the spin coating technique. The limit of detection for this sensing layer was observed to be 0.169 µM [81]. Another study of Co^2+^ detection by Saleviter et al. was also done. The distinction from the previous work was that they used cadmium sulfide quantum dot-graphene oxide–chitosan (CdS QDs-GO–CS) nanocomposite thin film. As reported, there was a shift in the resonance angle when the active layer interacted with deionized water and the concentration of Co^2+^ in a range of 0.169–169.68 µM [82].

**Figure 7:**
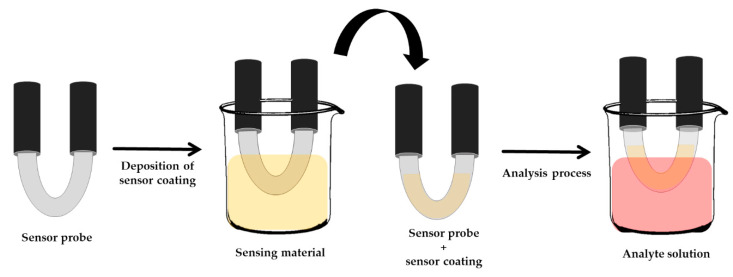
The development of a U-shaped probe for surface plasmon resonance [83].

Contradictorily with others, Boruah and Biswas developed an optical fiber-based surface plasmon resonance for Pb^2+^ detection. As shown in Figure 7, the U-shaped probe was dipped into the sensing material, a chitosan solution, for 5 min. After that, it was dried for 30 min at room temperature. Again, the sensor probe was dipped into 1 mM glutathione for 10 min and it was allowed to dry for 30 min. Next, the sensor probe was inserted in the analyte solution, and the calculated detection limit of 6.274 nM was obtained [83].

Abdullah et al. proposed a naturally-based kappa-carrageenan (κCarr) and chitosan (CS) using a localized surface plasmon resonance sensor to detect Pb^2+^. They fabricated the films by depositing 0.1 mL of κCarr and CS solutions on ITO/glass substrate covered by gold nanoparticles. They reported that AuNP–κCarr had a good linear response with a higher value of sensitivity compared to the AuNP–CS. This was owing to the amount of oxygen atoms on functional groups of kCarr that produced more binding sites for incorporation with Pb^2+^ ions [84].

In 2019, Anas et al. reported that chitosan/hydroxyl-functionalized graphene quantum dots (CS/HGQDs) showed potential in detecting Fe^3+^ ions. Based on the photoluminescence analysis, the thin films exhibited a blue color, and the photoluminescence intensity increased when the HGQDs combined with chitosan solution. The detection for Fe^3+^ ion, between 8.953 µM and 1.79 mM, showed a positive response with a higher value of sensitivity. The potential of graphene-based material combined with polymeric materials had been extensively developed [85]. Then, Ramdzan et al. used another functional group of graphene quantum dots, namely carboxyl-functionalized graphene quantum dots (CGQDs), to detect Hg^2+^. In the range from 2.493 to 498.529 µM of Hg^2+^, the sensing layer was reported to have a linear progression, which indicated a sensitivity of 124.366° M^−1^ [86].

A work by Roshidi et al. combined poly(amidoamine) (PAMAM) dendrimers and chitosan to develop a Pb^2+^ ion sensor. They observed there were slight shifts of resonance angle for Au/CS–PAMAM film in contact with deionized water and also several concentrations of Pb^2+^, ranging between 0.483 and 2.413 µM. This change might be attributed to differences in the refractive index and the thickness of the thin film [87].

On the other hand, fiber-optic surface plasmon resonance was also used for heavy metal ion detection by Ding et al. with gold film and chitosan/poly(acrylic acid) (CS/PAA). In order to prepare the sensor, no-core fiber (NCF) was coated with a gold film by ion beam sputtering followed by repetition of a simple dip-coating with CS/PAA to obtain an NCF sensor with ten bilayers. At a low concentration of Cu^2+^ ions, this functionalized sensor showed better performance; in the range of 3.147–786.832 µM, the sensitivity was 0.1184 M^−1^, compared to 0.0117 M^−1^ for a concentration range of 786.832–7.868 mM. They also reported the detection limit of Cu^2+^ was 0.1054 µM [88].

In the same year, reusable surface plasmon resonance was used by Wang et al. for the recognition of Cu^2+^. This time, the silver–gold (Ag/Au) film was used to enhance the stability and sensitivity of the SPR sensor. The Ag/Au film was coated with a modified-chitosan solution (MCS) by a spin coating method. From the results, the Ag/Au/MCS film had a detection limit of 0.283 µM with a sensitivity value of 46,204.30° M^−1^. Besides, a small error bar was obtained based on the graph of signal response against Cu^2+^ concentration, which was a good sign for the repeatability of the sensor [89].

Then, a work by Saleviter et al. for Co^2+^ detection using an SPR sensor was reported using chitosan–graphene oxide decorated quantum dots (CS–GO/CdS QDs) modified on a gold layer. There was no shift in the resonance angle when the gold thin film was used to detect different concentrations of Co^2+^ ranging from 0.169 nM to 1.696 mM. Then, when the gold thin film was replaced by CS–GO/CdS QDs, the detection limit of 0.169 µM was obtained. This sensing layer was more sensitive for lower concentrations due to the value of sensitivity being 7001.240° M^−1^ (0.169–16.968 µM). Meanwhile, for 16.968 µM to 169.684 µM, the sensitivity was 477.358° M^−1^ and 23.573° M^−1^ for the higher concentration of 169.684 µM to 1.696 mM [90].

Another study on graphene-based composites, Omar et al. prepared gold/cadmium sulfide quantum dot-reduced graphene oxide/antibodies (Au/CdSQD-rGO/Abs) and chitosan–graphene oxide–cadmium sulfide quantum dot (CS–GO–CdS QD) thin film as optical sensor chips in biomedical applications. These two thin films were used for the rapid and quantitative detection of dengue virus (DENV) and Co^2+^, respectively. The results showed that the resonance angle shifted to the right as the concentration of Co^2+^ increased. The CS–GO–CdS QDs thin film could detect as low as 1.697 µM of Co^2+^ with a high sensitivity value [91].

A recent work by Daniyal et al. used coated nanocrystalline cellulose modified with hexadecyltrimethylammonium bromide and graphene oxide (CTA/NCC/GO) on top of the gold film to detect Cu^2+^. The CTA/NCC/GO could detect Cu^2+^ as low as 157.366 µM, and the presence of CTA/NCC/GO on gold thin film also resulted in a high binding affinity constant of 4.075 × 10^3^ M^−1^ [92]. In the same year, by using the same active layer (CTA/NCC/GO), Daniyal et al. once again incorporated the sensing layer with an SPR to detect Ni^2+^ and Zn^2+^. The results showed that Ni^2+^ and Zn^2+^ could be detected as low as 0.170 µM and 0.153 µM, respectively [93,94].

### 3.2. Incorporation of Conducting Polymer with Surface Plasmon Resonance

Although biopolymer-based composites have been widely used in SPR sensors, researchers also study conducting polymer-based materials for heavy metal ion detection in aqueous solution. Interestingly, conducting polymers such as polypyrrole, polyaniline and polythiophene also enhance the sensitivity of the sensing layer in SPR sensors. In 2004, the first experiment of the SPR sensor using conducting polymers was studied by Yu et al. The chemical binding interaction between Hg^2+^ with polypyrrole (PPy) and 2-mercaptobenzothiazole (2-MBT) was monitored. At 49.853 µM, there were increases in the SPR angle of (780 ± 10) × 10^−40^, and in the range of 0.498 µM to 49.853 µM, a linear dynamic range was observed. The limit of detection was improved to 0.0498 µM after 2-MBT was injected into the polymer. It was concluded that 2-MBT acted as a sensitivity enhancement agent that produced further binding interaction for detecting Hg^2+^ bound on the PPy surface [95].

In 2012, Sadrolhosseini et al. synthesized polypyrrole thin film through an electrochemical method to deposit the sensing layer on a gold film and used it to detect Cu^2+^ and Fe^3+^. They found that the detection limit of the PPy sensing layer was about 1.574 µM, and the sensor was more sensitive to Cu^2+^ compared to Fe^3+^ [96]. Two years later, Sadrolhosseini et al. developed a polypyrrole multi-walled carbon nanotube (PPy-MWCNT) composite layer for detection of Hg^2+^, Pb^2+^ and Fe. As reported before, an MWCNT was used to enhance the sensitivity and accuracy of the sensor, such that the angle shift increased relative to the angle shift of PPy thin film to quantify the different concentrations of all-mentioned ions. The sensor showed a sensitive response to Hg^2+^ better than Pb^2+^ or Fe^3+^ with a detection limit of about 0.498 µM [97].

The fabrication of gold nanoparticles/graphene oxide/polyaniline (AuNPs/GO/PANI) nanocomposites film was done by Nawi et al. to detect Pb^2+^ using an LSPR-based sensor. The nanocomposite layer was prepared by loading 40–50 nm of AuNPs onto indium–tin oxide (ITO) glass. Then, a GO/PANI nanocomposite solution was deposited onto AuNP–ITO glass using a Laurell Technologies Corporation photoresist spinner. The AuNP/GO/PANI film was exposed to different concentrations of Pb^2+^ that varied from 0.145 to 14.479 µM. Thus, the sensing layer exhibited high sensitivity with a limit of detection as low as 0.145 µM [98].

In the following year, Bahrami et al. furthered the study on the PPy–MWCNT composite layer for detecting Al^3+^ in aqueous solution. In their study, two sensing layers, i.e., PPy–MWCNT and polypyrrole–chitosan (PPy–CS) layer, were compared based on the results of the measurement of the concentration of Al^3+^. The sensitivity of the PPy–MWCNT layer was higher than PPy–CS, and the limit of the sensors was about 3.706 µM [99].

Still in 2015, Tabassum and Guptaused surface plasmon resonance based fiber optics for the detection of manganese ions (Mn^2+^). In their project, the probe was prepared by depositing a nanocomposite of polypyrrole (PPy) and zinc oxide (ZnO) onto the silver-coated unclad core of the fiber. Then, the prepared fiber probe was interacted with the Mn^2+^ solution, and as the concentration of Mn^2+^ increased up to 0.2 M, the sensitivity seemed to decrease nonlinearly. Thus, this sensor was sensitive and selective to Mn^2+^, with the lowest detection limit of 0.673 µM [100].

As two is better than one, there were also studies on the integration of biopolymers and conducting polymers to enhance the sensitivity of the sensing layer to detect heavy metal ions using SPR. Sadrolhosseini et al. prepared a polypyrrole–chitosan (PPy–CS) sensing layer for the detection of Cu^2+^. Instead of using the spin coating method, electrochemical deposition was used in this study to deposit the sensing layer on a gold thin film. From the observations, the SPR signal showed a slight difference when the concentration of Cu^2+^ increased up 1.573 mM [101].

Still in 2011, the detection of Hg^2+^ and Pb^2+^ based on conducting polymer composites was done by Abdi et al. In this study, electrochemical deposition was also used to prepare the PPy–CS films. They reported that the resonance angle was changed when the concentration was varied in the range from 2.493 µM to 59.824 µM. By comparing the results, it could be seen that Pb^2+^ ion bonded to the PPy–CS composite films most strongly, and the sensor was generally more sensitive towards Pb^2+^ than Hg^2+^ [102]. In next year, Sadrolhosseini et al. used the same sensing layer in another focus to detect Fe^3+^ corrosion. This time, the range of concentration was 1.791 µM to 1.343 mM and the resonance angle was right-shifted. Thus, this sensing layer was able to detect Fe^3+^ as low as 1.791 µM [103].

In 2013, another work by Sadrolhosseini et al. attempted to apply PPy–CS as a sensing layer with SPR for Zn^2+^ and Ni^2+^ detection. Using concentrations of Zn^2+^ and Ni^2+^ ranging from 0.153 µM to 1.277 mM, a resonance angle shift was observed. The detection limits for the sensors of PPy–CS were about 0.153 µM and 0.170 µM for Zn^2+^ and Ni^2+^, respectively. They also reported that the Zn^2+^ had a higher affinity constant value of 2.301 × 104 M^−1^ compared to Ni^2+^ with 1.672 × 104 M^−1^ [104].

A work in 2015 by Verma and Gupta, developed an SPR sensor-based optical fiber sensor using conducting polymer and chitosan in heavy metal ion detection. They used silver (Ag) metal and indium tin oxide (ITO) as the base of the SPR probe, which was further modified with a coating of PPy–CS composite. The prepared sensor was used to detect Cd^2+^, Pb^2+^ and Hg^2+^ with concentration ranges up to 1.779 µM. From the observations, the detection limits of Cd^2+^, Pb^2+^ and Hg^2+^ were 1.29 nM, 1.58 nM and 2.93 nM, respectively. They also reported that PPy/CS/ITO/Ag showed a higher sensitivity when detecting Cd^2+^ compared to other heavy metal ions [105].

In 2017, Sadrolhosseini et al. stepped up the game by using a polypyrrole–chitosan/nickel–ferrite nanoparticle composite (PPy–CS/NiFe2O4–NPs) layer in SPR. The PPy–CS/NiFe2O4–NPs was deposited on gold-coated glass by the electrochemical method for further use in detecting Ni^2+^, Fe^3+^, Co^2+^, Al^3+^, Mn^2+^, Hg^2+^ and Pb^2+^. It could be seen that the detection limit of ferromagnetic ions (Ni^2+^, Fe^3+^, Co^2+^) was about 17.3 nM and that of paramagnetic ions (Al^3+^, Mn^2+^) was 37.1 nM, while for diamagnetic ions (Hg^2+^, Pb^2+^) the detection limit was 4.82 nM [106].

Another study in detecting arsenic was done by the same author, Sadrolhosseini et al. However, in this project, they used a different active layer, which was a polypyrrole–chitosan/cobalt–ferrite nanoparticle (PPy–CS/CoFe2O4–NPs) composite. The nanoparticle composite was deposited on a gold-coated glass slide by the electrodeposition method. In a range from 0.0133 to 1.334 mM, the SPR signals were recorded. Then, compared to the previous study of arsenic detection, the limitation of the sensor had improved to 13.3 nM [107]. The biopolymers and conducting polymers used for heavy metal ion detection by SPR are summarized in Table 2.

## 4. Future Perspectives in the Development of Biopolymers and Conducting Polymers with Surface Plasmon Resonance for Heavy Metal Ion Detection

Nowadays, there are tremendous efforts from researchers worldwide in developing sensors to overcome heavy metal ion contamination in water. The demand for sensitivity, selectivity and the ability to detect an analyte in very low concentrations is very high. Colorimetric, electrochemiluminescence, fluorescence and surface plasmon resonance have emerged as potential sensors for sensing heavy metal ions in aqueous solution. These optical sensors have different performances for sensing an analyte in terms of detection limit and sensitivity. The performance of these optical sensors for heavy metal ion detection is summarized in Table 3. The overview focused on the limit of detection, which is more significant in terms of comparison among the sensors.

Overall, the surface plasmon resonance technique has a rather higher detection limit of 1.29 nM. This is very much affected by the biopolymers/conducting polymers that are introduced to incorporate with the optical sensor [108]. To further increase the sensitivity, a better combination of biopolymer and conducting polymer can be explored. A novel composite material with improved sensing efficiency should be attentive to lower the limit of detection. Accordingly, it is believed that polythiophene–nanocrystalline cellulose has great potential to be developed as a sensing layer. Significant future potential is due to polythiophene having higher optical transparency compared to other conducting polymers [109]. This physical property will ensure the surface plasmon resonance phenomena occurs effectively. By adding poly(styrenesulfonate), polythiophene is more stable and convenient to fabricate sensing thin film with other polymers. Furthermore, polythiophene has aqueous compatibility and biocompatibility to incorporate with biopolymer materials and has a higher charge capacity compared to polypyrrole-based materials [110]. Meanwhile, nanocrystalline cellulose boasts favorable properties, i.e., high adsorption capacity, hydrophilicity and changeable appearance. Thus, the large amount of OH bonds also ensure the stability of the structure of blended materials [111]. Thus, by integrating novel potential biopolymers and conducting polymers with surface plasmon resonance, the ability to detect heavy metal ions can be enhanced.

Hence, further research could be extended in designing biopolymers and conducting polymer-based materials to improve the sensitivity and selectivity of the optical sensor. Environmental sensors can be developed from the integration of these polymers to help in diminishing heavy metal ion contamination. Furthermore, the potential development of sensing materials to be implemented into Biacore 8K, SPR-based biosensors can also be looked forward to. Like our prediction, the demand for surface plasmon resonance-based sensors will increase in the future as they have potential in a wide range of applications.

## 5. Conclusions

As it has been chronologically outlined, the development of biopolymers and conducting polymer-based sensors in recognition of heavy metal ions was reviewed in this paper. In this review, a summary of the potential application of SPR sensors using several types of biopolymers and conducting polymers with different analytes was also discussed. With impressive and convincing properties, various types of polymers have been extensively applied in sensing applications. As a conclusion, the invention of biopolymers and conducting polymers with SPR sensors can be considerable to develop novel sensing composites with excellent sensitivity and selectivity for heavy metal ion detection and other real sensing applications in the future.

## Figures and Tables

**Table 1:** The biopolymers and conducting polymer-based optical sensors for heavy metal ion detection.

Metal Ion	Type of Polymers	Optical Sensors	Linear Range	Limit of Detection	References
Zn^2+^	Chitosan modified with 5-formyl-3-hydroxy-4-hydroxymethyl-2-methylpyridine	Fluorescence	0–25 µM	Zn^2+^: 1 µM	[27]
Cd^2+^				Cd^2+^: 1 mM	
Ga^3+^				Ga^3+^: 1 mM	
Cu^2+^	Cadmium sulfide quantum dots modified by chitosan	Fluorescence	8 nM–3 μM	1.2 nM	[28]
Hg^2+^	Chitosan nanoparticles	Fluorescence	0–1 mM	1 µM	[29]
Cu^2+^	Magnetic and fluorescent bifunctional chitosan nanoparticles	Fluorescence	1.967–393.416 µM	0.724 µM	[30]
K^+^	Chitosan/Ru(bpy)_3_^2+^/silica nanoparticle-modified glass carbon electrode	Electrochemiluminescence	0–9 nM	0.3 nM	[31]
Hg^2+^	Three-dimensional fluorescent chitosan hydrogel	Fluorescence	5.0–50 nM	0.9 nM	[32]
Hg^2+^	Chitosan-functionalized gold nanoparticles	Colorimetric	0.05–9 µM	1.35 µM	[33]
Hg^2+^	Chitosan thiomer	Colorimetric	0–498.5 µM	2.318 µM	[34]
Hg^2+^	Chitosan microspheres	Fluorescence	0.5–7 µM	0.015 µM	[35]
Hg^2+^	Chitosan–rhodamine	Fluorescence	0–6 µM	3.42 µM	[36]
Cd^2+^	Chitosan dithiocarbamate functionalized gold nanoparticles	Colorimetric	0.1–500 µM	0.063 µM	[37]
Hg^2+^	Chitosan–silver nanocomposite	Colorimetric	0–500.870 µM	7.2 nM	[38]
Cu^2+^	Carbon dot-embedded fluorescent magnetic nanoparticles O-carboxymethyl chitosan	Fluorescence	0.01–200 µM	0.56 µM	[39]
Hg^2+^	Chitosan/silver–gold nanocomposite	Colorimetric	-	0.5 nM	[40]
Hg^2+^	Chitosan stabilized gold nanoparticles and 2,6-pyridinedicarboxylic acid	Colorimetric	0.3–5 µM	-	[41]
Al^3+^	Chitosan/silver	Fluorescence	2–180 µM	-	[42]
Cu^2+^	Tetraphenylethylene–chitosan	Fluorescence	5–100 µM	-	[43]
Cu^2+^	Chitosan–Fluorescein isothiocyanate oligomer	Colorimetric	0.1 µM–7 mM	60 µM	[44]
Hg^2+^	Chitosan–gold nanoparticles	Colorimetric	0–13.6 µM	0.02 µM	[45]
Hg^2+^	Silver-doped cadmium sulfide quantum dots/chitosan	Colorimetric	124–800 µM	124 µM	[46]
Hg^2+^	Thiol terminated chitosan capped silver nanoparticles	Colorimetric	0–0.4 µM	0.017 µM	[47]
Hg^2+^	Chitosan functionalized gold nanoparticles assembled on Sulphur doped graphitic carbon nitride	Colorimetric	0.1–0.5 µM	0.275 nM	[48]
Hg^2+^	Chitosan–gold nanocomposite	Colorimetric	0–2 µM	3.2 nM 0.05 µM	[49]
Ag^+^	Chitosan functionalized gold nanoparticles	Colorimeter	1–100 µM	0.13 µM	[50]
Zn^2+^	Cellulose film modified with 1-(2-pyridylazo)-2-naphthol (PAN)	Colorimetric	10–100 µM	-	[51]
Mn^2+^					
Ni^2+^					
Hg^2+^	Ruthenium dye or mercaptosilane monolayer onto metal oxide ultrathin film pre-coated cellulose nanofibers	Colorimetric	0.050–100 µM	0.049 µM	[52]
Cr^3+^	1,4-DHAQ-doped cellulose nanofiber Films	Fluorescence	Cu^2+^: 2.5–37.5 nM	-	[53]
Cr^3+^			Cr^3+^: 2.5–25 nM		
Fe^3+^	Pyrene–cellulose nanocrystals	Fluorescence	0–5 mM	1 µM	[54]
Pb^2+^	Pyromellitic dianhydride-grafted cellulose nanofibrous membranes	Colorimetric	0.048–5 µM	0.048 µM	[55]
Hg^2+^	Au nanoparticles/carboxylethyl quaternized cellulose	Colorimetric	0–0.3 µM	0.04 µM	[56]
Hg^2+^	Cellulose–rhodamine	Fluorescence	0–10 mM	50 µM	[57]
Hg^2+^	Cellulose–Lysine Schiff-Base	Fluorescence	49.853–498 µM	49.853 µM	[58]
Fe^2+^	1,10-phenanthroline-5-amine-4-4′-methylene diphenyl diisocyanate-cellulose acetate	Fluorescence	0–17.8 µM	46.6 nM 0.895 µM	[59]
Pb^2+^	Nanocrystalline cellulose-ethylenediamine	Fluorescence	18.7 nM–0.5 µM	0.024 µM	[60]
Cu^2+^	Rhodamine-B derivative and bacterial cellulose	Colorimeter	4–128 µM	-	[61]
Fe^3+^	Curcumin embedded bacterial cellulose	Colorimetric	0.01–100 µM	7.8 µM 8.2 µM	[62]
Cu^2+^	Polyaniline/polyamide-6 nano-fiber/net membranes	Colorimetric	0–1573 µM	15.737 µM	[63]
Hg^2+^	Polyaniline leucoemeraldine base nanofibrous	Colorimetric	0–150 µM	0.005 µM	[64]
Hg^2+^	Polyaniline/carbon dot nanocomposites	Fluorescence	0.05–1 µM	0.8 nM	[65]
Hg^2+^	Polyaniline microspheres	Fluorescence	0–1.5 µM	0.86 nM	[66]
As^3+^	Polyaniline nanofiber/carbon dot nanohybrid	Fluorescence	0–0.026 µM	1.3 pM	[67]
Fe^3+^	Nanostructured polypyrrole film doped Tiron	Fluorescence	0.05–1 µM	0.01 µM	[68]
Pb^2+^	Benzene sulfonic acid doped polypyrrole	Fluorescence	0–9 µM	Pb^2+^: 0.018 µM	[69]
Cu^2+^				Cu^2+^: 3.1 nM	

**Table 2:** The biopolymers and conducting polymers based on surface plasmon resonance for metal ion detection.

Metal Ion	Type of Polymers	Linear Range	Limit of Detection	References
Fe^3+^	Chitosan thin film	4.477 µM–1.79 nM	4.477 µM	[58]
Cr^6+^	Gold nanoparticles/chitosan composite	192.322 µM–4.808 nM	192.322 µM	[71]
Hg^2+^	Chitosan cross-linked glutaraldehyde solution	Hg^2+^: 2.493–498.529 µM	Hg^2+^: 2.493 µM	[72]
Cu^2+^		Cu^2+^: 7.868 µM–1.573 nM	Cu^2+^: 7.868 µM	
Cu^2+^	Chitosan cross-linked glutaraldehyde solution	Cu^2+^: 7.868 µM–1.573 mM	Cu^2+^: 7.868 µM	[73]
Zn^2+^		Zn^2+^: 7.648 µM–1.529 mM	Zn^2+^: 7.648 µM	
Mn^2+^		Mn^2+^: 9.101 µM–1.820 mM	Mn^2+^: 9.101 µM	
Pb^2+^	Immobilized p-tert-butylcalix[4]arene-tetrakis in chitosan thin film	0.144–24.131 µM	0.144 µM	[74]
Pb^2+^	Crosslinked chitosan	2.413–482.625 µM	2.413 µM	[75]
Pb^2+^	Gold–chitosan–graphene oxide nanostructured thin films	0.145–24.131 µM	0.145 µM	[76]
Zn^2+^	Chitosan–tetrabutyl thiuram disulfide	0.153–76.476 µM	0.153 µM	[77]
Pb^2+^	Chitosan–graphene oxide	0.483–24.131 µM	0.483 µM	[78]
Pb^2+^	Gold/silver/gold/chitosan–graphene oxide	Pb^2+^: 0.483–24.131 µM	Pb^2+^: 0.483 µM	[79]
Hg^2+^		Hg^2+^: 0.499–24.926 µM	Hg^2+^: 0.499 µM	
K^+^	Valinomycin doped chitosan–graphene oxide thin film	0.0256 µM–2.557 mM	0.0256 µM	[80]
Co^2+^	Immobilized 4-(2-pyridylazo) resorcinol in chitosan–graphene oxide composite thin film	0.169 µM–1.696 mM	0.169 µM	[80]
Co^2+^	Cadmium sulfide quantum dots–graphene oxide–chitosan nanocomposite thin film	0.169–169.684 µM	0.169 µM	[82]
Pb^2+^	Chitosan–glutathione coated sensor probe	4.826–33.784 µM	6.274 µM	[83]
Pb^2+^	Gold nanoparticle kappa-carrageenan and chitosan	0.048–24.131 µM	0.048 µM	[84]
Fe^2+^	Chitosan/hydroxyl-functionalized graphene quantum dots thin film	8.953 µM–1.79 mM	8.953 µM	[85]
Hg^2+^	Chitosan/carboxyl-functionalized graphene quantum dots thin film	2.493–498.529 µM	2.493 µM	[86]
Pb^2+^	Chitosan–poly(amidoamine) dendrimer composite thin film	0.483–2.413 µM	0.483 µM	[87]
Cu^2+^	Chitosan/poly(acrylic acid) bilayers	3.147 µM–7.868 mM	0.1054 µM	[88]
Cu^2+^	Silver/gold composite film modified by modified-chitosan (MCS) thin film	15.737–78.683 µM	0.283 µM	[89]
Co^2+^	Chitosan–graphene oxide/cadmium sulphide quantum dots active layer	0.169 µM–1.696 mM	0.169 µM	[90]
Co^2+^	Chitosan–graphene oxide–cadmium sulfide quantum dots composite thin film	1.697 µM–1.696 mM	1.697 µM	[91]
Cu^2+^	Nanocrystalline cellulose modified by hexadecyltrimethylammonium bromide and graphene oxide composite thin film	157.366–944.198 µM	157.366 µM	[92]
Ni^2+^	Nanocrystalline cellulose–graphene oxide-based nanocomposite	0.170–170.378 µM	0.170 µM	[93]
Zn^2+^	Modified-nanocrystalline cellulose/graphene oxide	0.153–152.952 µM	0.153 µM	[94]
Hg^2+^	Polypyrrole and 2-mercaptobenzothiazole	0.498–49.853 µM	0.0498 µM	[95]
Cu^2+^	Polypyrrole thin film	Cu^2+^: 1.574–314.733 µM	Cu^2+^: 1.574 µM	[96]
Fe^3+^		Fe^3+^: 1.791–358.134 µM	Fe^3+^: 1.791 µM	
Hg^2+^	Polypyrrole multi-walled carbon nanotube composite layer	Hg^2+^: 0.498–498.529 µM	Hg^2+^: 0.498 µM	[97]
Pb^2+^		Pb^2+^: 0.483–482.625 µM	Pb^2+^: 0.483 µM	
Fe^2+^		Fe^2+^: 1.791 µM–1.79 mM	Fe^2+^: 1.791 µM	
Pb^2+^	Gold nanoparticles/graphene oxide/polyaniline nanocomposites film	0.145–14.479 µM	0.145 µM	[98]
Al^3+^	Polypyrrole multiwalled carbon nanotube composite layer	3.706 µM–3.706 mM	3.706 µM	[99]
Mn^2+^	Nanocomposite of polypyrrole and zinc oxide over silver	0–0.2 M	0.673 µM	[100]
Cu^2+^	Polypyrrole–chitosan layer	1.574 µM–1.573 mM	1.574 µM	[101]
Hg^2+^	Polypyrrole–chitosan conducting polymer composite	2.493 µM–59.824 µM	2.493 µM	[102]
Pb^2+^				
Fe^3+^	Polypyrrole–chitosan layer	1.791 µM–1.343 mM	1.791 µM	[103]
Zn^2+^	Polypyrrole–chitosan	Zn^2+^: 0.153–59.824 µM	Zn^2+^: 0.153 µM	[104]
Ni^2+^		Ni^2+^: 0.170 µM–1.277 mM	Ni^2+^: 0.170 µM	
Cd^2+^	Polypyrrole and chitosan/ITO/silver	-	Cd^2+^: 1.29 nM	[105]
Pb^2+^			Pb^2+^: 1.58 nM	
Hg^2+^			Hg^2+^: 2.93 nM	
Ni^2+^ Fe^2+^ Co^2+^ Al^2+^ Mg^2+^ Hg^2+^ Pb^2+^	Polypyrrole–chitosan/nickel–ferrite nanoparticle composite layer	-	Ni^2+^, Fe^2+^, Co^2+^: 17.3 nM Al^2+^, Mg^2+^: 37.1 nM Hg^2+^, Pb^2+^: 4.82 nM	[106]
V	Polypyrrole–chitosan–cobalt ferrite nanoparticles composite layer	0.0133–1334.721 µM	13.3 nM	[107]

**Table 3:** The comparison of the limit of detection of optical sensors.

Optical Sensor	Biopolymers/Conducting Polymers	Lowest Limit of Detection	Heavy Metal Ion	Reference
Electrochemiluminescence	Chitosan/Ru(bpy)_3_^2+^/silica nanoparticle-modified glass carbon electrode	0.3 nM	K^+^	[31]
Colorimetric	Chitosan functionalized gold nanoparticles assembled on sulphur doped graphitic carbon nitride	0.275 nM	Hg^2+^	[48]
Fluorescence	Polyaniline nanofiber/carbon dot nanohybrid	1.3 pM	As^3+^	[67]
Surface plasmon resonance	Polypyrrole–chitosan/ITO/silver	1.29 nM	Cd^2+^	[104]
